# stam – a Bioconductor compliant R package for structured analysis of microarray data

**DOI:** 10.1186/1471-2105-6-211

**Published:** 2005-08-25

**Authors:** Claudio Lottaz, Rainer Spang

**Affiliations:** 1 Max Planck Institute for Molecular Genetics and Berlin Center for Genome Based Bioinformatics, Ihnestr. 73, D-14195 Berlin, Germany

## Abstract

**Background:**

Genome wide microarray studies have the potential to unveil novel disease entities. Clinically homogeneous groups of patients can have diverse gene expression profiles. The definition of novel subclasses based on gene expression is a difficult problem not addressed systematically by currently available software tools.

**Results:**

We present a computational tool for semi-supervised molecular disease entity detection. It automatically discovers molecular heterogeneities in phenotypically defined disease entities and suggests alternative molecular sub-entities of clinical phenotypes. This is done using both gene expression data and functional gene annotations.

We provide stam, a *Bioconductor *compliant software package for the statistical programming environment *R*. We demonstrate that our tool detects gene expression patterns, which are characteristic for only a subset of patients from an established disease entity. We call such expression patterns molecular symptoms. Furthermore, stam finds novel sub-group stratifications of patients according to the absence or presence of molecular symptoms.

**Conclusion:**

Our software is easy to install and can be applied to a wide range of datasets. It provides the potential to reveal so far indistinguishable patient sub-groups of clinical relevance.

## Background

Microarray analysis is among the most promising clinical applications of modern genomics. It opens perspectives for more reliable and efficient diagnosis of established tumor entities [1,2], risk group determination [3,4], and the prediction of response to treatment [5]. In the supervised setting, various software tools implementing algorithms from statistical learning theory are available and have been evaluated in the context of microarray data (e.g. [6-10]).

All these methods aim for reproducing or predicting predefined clinical phenotypes. However, often clinical phenotypes will not be homogeneous from a molecular point of view. For example, when distinguishing between recurrent and non-recurrent disease, it is of course possible that recurrence has various molecular backgrounds. If this is the case, one will expect different molecular changes in different patients, and purely supervised analysis is unsatisfactory.

In several studies, unsupervised clustering algorithms have been applied to patient profiles, with the aim to define novel disease entities [11-14]. However, clustering of patients is not straightforward, since the clinical relevance of a clustering result is often unclear. It is quite possible that a given clustering reflects unimportant covariates like gender and age or even experimental artifacts. This is usually avoided by visual inspection of the clustered data and an educated manual selection of interesting genes. Automated software tools for this problem are not available so far.

We have recently suggested a novel algorithm for semi-supervised analysis called *structured analysis of microarrays *[15]. We consider the setting where a disease group is to be distinguished from a set of patients with a different clinical phenotype (controls). Instead of determining a single global signature to detect all disease cases, we generate several local signatures, which identify only subsets. We call the local signatures *molecular symptoms*. A special feature of the method is that it produces multiple candidate symptoms and characterizes each by a functional annotation, like patients with poor prognosis and altered expression of apoptosis related genes. The functional annotations stored in the Gene Ontology (GO) are used to ensure biological focus.

In GO [16], terms describing biological processes, molecular functions and cellular localizations are organized in a directed acyclic graph, where each node represents a biological process and child-terms are either members or representatives of their parent-terms. Genes are attributed to nodes according to the knowledge the biological research community has gathered so far. Molecular symptoms found by stam exclusively contain genes associated with one node of the Gene Ontology and therefore have a biological focus.

**Figure 1:**
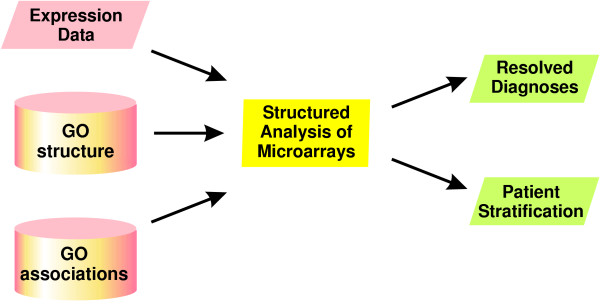
**Overview on structured analysis of microarrays**. Input of stam is a gene expression dataset, the structure of the Gene Ontology and associations of genes with GO terms. The output of the method is a resolved diagnosis per patient according to molecular symptoms, arid thus a molecular stratification of patients according to absence and presence of these symptoms.

For each patient and each node classifier stam calculates a value between 0 and 1 for each relevant molecular symptom. These values indicate how likely it is that a patient belongs to the disease class according to the corresponding molecular symptom. In addition, we suggest to use patterns of absence or presence of molecular symptoms to stratify patients into subclasses. An overview of the algorithm is given in Figure 1.

## Implementation

A detailed description of structured analysis of microarrays is given in [15]. Here we only give a brief review of the method.

**Figure 2:**
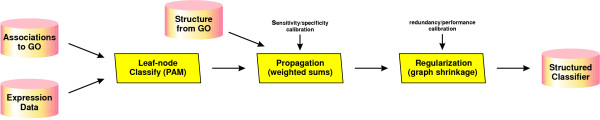
**stam's algorithm in a nutshell**. The algorithm for *structured analysis of microarrays *splits into three phases: classification in leaf nodes, propagation through inner nodes and shrinkage of the classifier graph. Calibration by the user allows for fine-tuning of specificity versus sensitivity in classifier evaluation and redundancy versus performance tradeoff in graph shrinkage.

We use Gene Ontology's hierarchical structure. Based on the GO graph of biological terms, stam generates a classifier graph holding one classifier for each node. The classifiers only depend on genes annotated to corresponding nodes or their descendants. In a nutshell, stam consists of the following steps as illustrated in Figure 2:

• generate a rooted, directed classifier graph according to the gene Ontology,

• construct leaf-node classifiers based on expression values of genes, which are directly annotated to the leaf nodes,

• propagate the results through inner nodes to the root,

• and shrink the classifier graph to determine a concise set of molecular symptoms.

We have implemented the algorithm based on the R package for statistical computing [17]. Time-consuming parts of the method are written in C to improve computational performance. Furthermore, we rely on packages from the Bioconductor suite of bioinformatics tools [18].

### The raw classifier graph

Starting from a node of interest specified by the user, stam generates a graph of classifiers according to the structure of the Gene Ontology. The graph is generated anew for each chip type. Any GO node can be chosen to start the procedure with this node as root of the graph. The default is the root of the biological process branch of the gene ontology. Our implementation uses Bioconductor meta-data packages to obtain chip-specific associations of probe-sets with genes as well as the generic GO structure. Table 1 summarizes the annotation data, which is currently available for Affymetrix-GeneChip^® ^microarrays.

**Table 1:** Gene Ontology annotations available in Bioconductor – For the microarrays listed in this table, Bioconductor meta data packages are available. The second column gives the number of leaf nodes the third column the number of inner nodes considered when generating classifier graphs. The last column reports the ratio of probe-sets being associated with any leaf node.

	Species	Probe-sets	Leaf nodes	Inner nodes	Annotated
hgu133a	human	22283	1649	1049	67.6%
hgu133b	human	22645	1136	783	30.7%
hgul33plus2	human	54675	1725	1094	44.1%
hu6800	human	7129	1300	872	84.7%
hgu95av2	human	12625	1492	966	76.2%
hgu95b	human	12620	972	669	33.3%
hgu95c	human	12646	895	633	27.7%
hgu95d	human	12644	866	603	22.9%
hgu95e	human	12639	935	641	32.1%
mgu74av2	mouse	12488	1379	934	63.5%
mgu74bv2	mouse	12411	975	696	33.7%
mgu74cv2	mouse	11934	826	590	26.0%
moe430a	mouse	22690	1538	1017	61.4%
moe430b	mouse	22575	997	719	21.0%
rgu34a	rat	8799	974	689	46.0%
rgu34b	rat	8791	403	332	7.7%
rgu34c	rat	8789	439	345	8.5%
rae230a	rat	15866	1032	718	23.5%
rae230b	rat	15276	319	297	3.1%
yg98	yeast	6777	1028	667	80.8%
YEAST	yeast	5799	1030	668	99.9%

### Leaf-node classifiers

Each leaf node contains a set of associated genes. The classifiers for leaf nodes are constructed using only these genes. For each patient, it returns a number between zero and one. Zero indicates clear evidence for the control group, one indicates clear evidence for the disease group and intermediate values represent levels of uncertainty. In the current implementation we use the shrunken centroid classifiers [9] implemented in the Bioconductor package pamr for leaf node predictions.

### Propagation of classifier results

For propagating leaf node results to inner nodes, weighted sums of child classifications are used. Children with good classification performance receive more weight than those with poor performance. Thereby, stam measures performance according to the desired properties of molecular symptoms by punishing low specificity more severely than lack of sensitivity. Prediction results are propagated from the leaf nodes towards the root in a postorder traversal of inner nodes. Hence, stam always computes results for all children before it computes results for the parent node. The root naturally displays an overall classification result.

### Classifier graph shrinkage

Many biological processes are not involved with the investigated phenotype. Therefore, stam simplifies the classifier graph by eliminating irrelevant branches. This is done in analogy to gene shrinkage in the shrunken centroid algorithm [9]. stam controls the shrinkage process by calibrating a shrinkage parameter in a cross validation setting. We define an objective function considering two independent goals: good predictive performance in the root and a set of molecular symptoms for patient stratification. For the second goal aggressive shrinkage is counterproductive, since it eliminates too many inherently heterogeneous molecular symptoms.

The program's output is a classifier graph, where each node represents a molecular symptom. We have shown in [15] that the collection of these classifiers yields state-of-the-art predictive performance and allows for a resolved diagnosis. A stam-diagnosis is more resolved than the classification provided for training because molecular symptoms are usually absent in some of the disease patients. Patterns of absence and presence of molecular symptoms identify smaller groups of patients and thus provide an additional molecular stratification of patients. Due to this unsupervised aspect within our supervised method, we call our approach semi-supervised.

## Results

Installing stam works like any other Bioconductor package either by downloading and installing from a local copy or directly through the internet. We provide packaged versions ready for download on the Bioconductor web site [19] as well as on our own web page [20].

Computing with stam is done on a command-line level. Gene expression matrices can either be provided as plain R matrices or as exprSet Bioconductor objects. R can read tab-delimited files written by any other software. stam provides functions for cross validation, model fit, and prediction. First, cross validation is applied on training data to find the appropriate shrinkage level. The second function computes a classifier model given this shrinkage level. This model can than be used by the prediction function to diagnose new patients and assign them to novel molecular disease entities. For convenience all three steps can be performed by one call of an evaluation function. This function can also randomly split patients into a training and a test set.

For further illustration, we use a data set from a microarray study on lung cancer [1]. The investigators have analyzed gene expression profiles from 186 lung cancer as well as 17 non-tumor lung biopsies using hierarchical and probabilistic clustering with the goal to uncover novel molecular lung cancer entities. The study uses the HG-U95Av2 microarray from Affymetrix and contains samples from various subtypes of lung cancer. For illustration, we apply stam with the squamous cancers forming the disease group of interest and all other cancers as controls. In the dataset there are 21 squamous carcinomas. The 203 samples are randomly split into a training set (135 samples containing 14 squamous) and a test set (68 samples containing 7 squamous).

### Automatic and manual calibration of graph shrinkage

**Figure 3:**
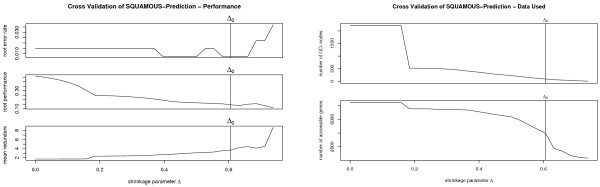
**Cross validation evaluation**. For several graph shrinkage candidates error rates in the root node, the root performance and the mean redundancy (top panel), as well as the number of nodes remaining in the shrunken graph and the number of genes accessible through these (bottom panel) are shown for the task to identify squamous lung cancers. The vertical lines show the automatically chosen shrinkage level.

**Figure 4:**
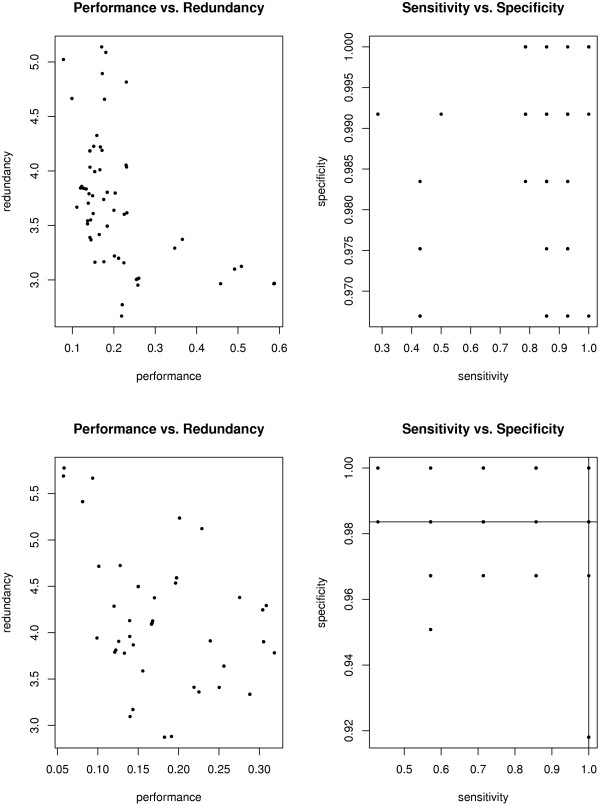
**Nodewise evaluation**. Classifiers for all nodes representing molecular symptoms have different performance. The left panels oppose performance to redundancy (to all other nodes remaining in the shrunken classifier graph). The right panels contrast sensitivity to specificity. The upper panels are drawn using training data while the lower ones are generated based on the test data.

Graph shrinkage can be calibrated automatically by cross validation or manually. To this end, stam provides two performance scores and corresponding plots. The first score is root performance measured as what would be the log-likelihood in a probabilistic setting. The second score, called mean redundancy, represents the diversity of molecular symptoms in the graph. It is the mean of pairwise redundancies. Here as well we interpret classifier outputs as probabilities. Our definition of pairwise redundancy is then the negative logarithm of the probability for unequal class prediction, stam aims for small values for both scores. While automated optimization uses an affine combination of the scores as objective function [15], manual calibration allows for a problem specific adjustment of the performance versus diversity trade off. The left pane of Figure 3 displays both scores for the whole classifier graph together with the error rate in the root node depending on the graph shrinkage level. The right pane of Figure 3 shows the number of nodes in the graph, and the number of genes accessible through these nodes. Figure 4 resolves the scores node per node. It contains scatter plots displaying sensitivity versus specificity and redundancy versus performance of single nodes. The redundancy of single nodes is defined in [15]. It is high if the corresponding classifier provides results which are similar to those of other classifiers in the shrunken graph.

### Browsable results

Results are written on interlinked HTML pages. Links allow navigation along the edges of the classifier graph. The pages contain classification results and performance evaluation for all nodes as well as overall information on cross-validation, model fit and root diagnosis of patients. For inner nodes the propagation weights are provided and for leaf nodes the genes used for classification can be displayed. The user can further explore term definitions and probe-set annotations through external links to the Gene Ontology and the Affymetrix web sites.

**Figure 5:**
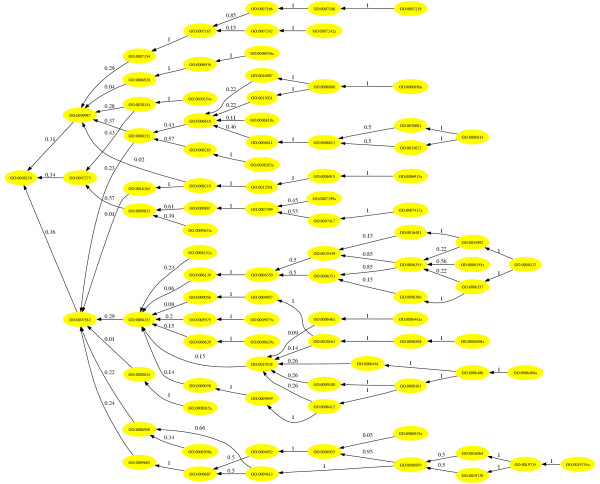
**Shrunken classifier graph**. For the squamous lung cancer identification this classifier graph containing 90 nodes is generated. Its 24 leaf nodes use 1614 probes (12.8% of all probes on the HG-U95Av2 microarray).

If the package graphviz [21] is installed, an interactive graphical representation of the classifier graph is included. Links on the nodes lead to the corresponding node-specific result page. Figure 5 shows an example using the lung cancers dataset.

**Figure 6:**
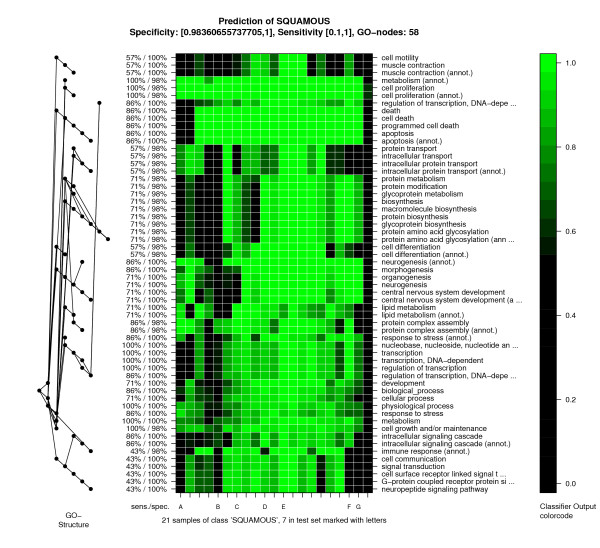
**Molecular symptoms image**. Resolved diagnoses for all squamous lung cancers are represented by the columns in this image. Rows represent molecular symptoms. The seven samples from the test set are marked with capital letters on the x-axis. Only a few samples including the test sample E present all molecular symptoms. Several samples lack some of the molecular symptoms. For instance, the symptom attributed to "intra-cellular protein transport" is not present in some of the training samples as well as samples B, C, F and G from the test set.

For visualizing patient stratification a molecular symptoms image is generated, as illustrated in Figure 6. Classifier outputs are color coded with bright colors representing presence, and dark color absence of a molecular symptom. Columns represent patients, and rows molecular symptoms. Rows and columns are arranged such that similar rows and columns are placed together.

### Interactive use of stam

**Figure 7:**
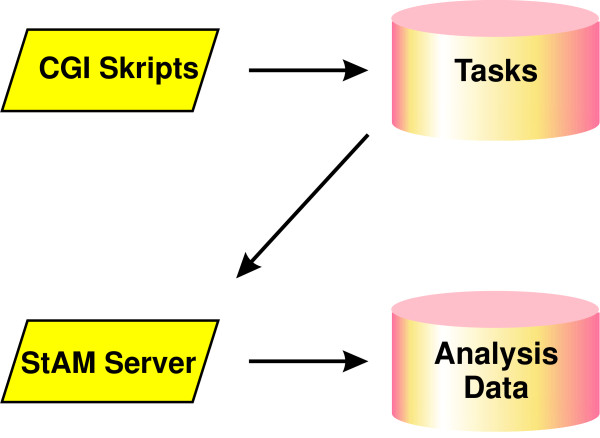
**Client-server architecture for interactive exploration**. Architecture of stam's server feature. CGI scripts deposit requests in a task repository from where the stam-server reads and executes them.

For the interactive exploration of the graph shrinkage level and other parameters affecting visual output, we have implemented a WWW based solution. stam can write HTML forms for these parameters directly on the HTML result pages. We provide CGI scripts with the package, which collect the user's entries and pass them to the stam server. This server is also included in the R package. It consists of an R function which, is constantly polling for user requests submitted via the internet. The architecture is illustrated in Figure 7. The user's WWW browser is redirected to a progress page which reloads automatically every second. As soon as the stam server has finished treating the request, the browser is redirected to an updated result page.

## Discussion

In this paper we present a software package to integrate biological annotation into statistical class prediction analysis of microarray data in an a priori fashion. We use the functional annotation collected in the Gene Ontology database to construct structured classifiers. Class predictions are computed for each term in the Gene Ontology which is related to the disease. Our method allows for biologically resolved diagnosis of patients. It is thus able to stratify complex clinical phenotypes, where different patients who show the phenotype may display different molecular characteristics.

We suggest structured analysis of microarrays for different applications. In addition to predictive performance we also aim for making underlying disease mechanisms transparent. We do this by identifying molecular symptoms associated to subsets of patients in the disease group. Molecular symptoms are always restricted to well defined biological processes. Patients who are positive for a molecular symptom display specific gene expression in the corresponding process. Not all patients in the disease group are positive for every identified molecular symptom, but some patients can be positive for more than one of them. Using patterns of absence and presence of molecular symptoms, we define an additional molecular stratification of patients.

## Conclusion

In summary, stam is a novel algorithm for uncovering previously unknown molecular disease sub-entities. The R package is easily accessible to all researchers working with Affymetrix^® ^oligo chips.

## Availability and requirements

The Bioconductor compliant R package stam is available through the Bioconductor web site [19]. Alternatively we also make it available on the Computational Diagnostics Software Page at the Max Planck Institute for Molecular Genetics in Berlin [20]. There, the source package is available for download and we also run a Bioconductor compliant package repository.

Our software is written for the R package for statistical computing downloadable from [22]. An installation of version 2.0.0 or later of the R software is needed to run stam. Our software is based on other Bioconductor packages, namely the meta data packages for the Gene Ontology annotations. We recommend to install release 1.5 of the Bioconductor suite from [19]. For the layout of classifier graphs, we rely on the graphviz package versions 1.10 or later available at [23].

We have extensively used stam on Linux installations on i686 based machines as well as alpha based UNIX machines running OSF1 and True64 operating systems.
